# Overexpression of Substance P in pig airways increases MUC5AC through an NF‐kβ pathway

**DOI:** 10.14814/phy2.14749

**Published:** 2021-02-13

**Authors:** Mariana Sponchiado, Yan‐Shin Liao, Kalina R. Atanasova, Emily N. Collins, Veronica Schurmann, Laura Bravo, Leah R. Reznikov

**Affiliations:** ^1^ Department of Physiological Sciences University of Florida Gainesville FL USA; ^2^ Department of Medicinal Chemistry and Center for Natural Products Drug Discovery and Development University of Florida Gainesville FL USA

**Keywords:** MUC5AC, MUC5B, neuropeptides, tachykinins

## Abstract

Substance P (SP) is a tachykinin that regulates airway mucous secretion in both health and disease. Our study aimed to determine whether overexpression of SP without pre‐existing inflammation was sufficient to induce changes in mucin secretion and transport in small airways. Utilizing porcine precision‐cut lung slices, we measured the impact of AAV‐mediated overexpression of SP on airway physiology ex vivo. Immunofluorescence signal intensity for MUC5AC was significantly increased in SP‐overexpressed precision‐cut lung slices compared to GFP controls. No difference in MUC5B signal intensity between treatments was detected. SP‐overexpressed precision‐cut lung slices also exhibited decreased *IL10 mRNA*, an important inhibitor of mucous cell metaplasia. Overt deficits in mucociliary transport were not noted, though a trend for decreased mean transport speed was detected in methacholine‐challenged airways overexpressing SP compared to GFP controls. Pharmacologic inhibition of the NF‐kβ pathway abrogated the effects of overexpression of SP on both MUC5AC and IL10. Collectively, these data suggest that overexpression of SP in the absence of existing inflammation increases MUC5AC via activation of the NF‐kβ pathway. Thus, these data further highlight SP as a key driver of abnormal mucous secretion and underscore NF‐kβ signaling as a pathway of potential therapeutic intervention.

## Graphical abstract

Overexpression of Substance P increased MUC5AC in porcine small airways in the absence of inflammation. The effect was mitigated by the inhibition of the NF‐KB pathway.

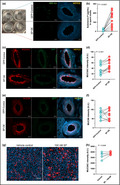

## 1 INTRODUCTION

Airway mucus hypersecretion is a hallmark feature in chronic life‐threatening respiratory conditions, such as asthma, cystic fibrosis (CF), and chronic obstructive pulmonary disease (COPD). Abnormal mucus production, secretion or clearance have deleterious consequences for airway function and health, including mucus stasis, augmented susceptibility to airway infection, and airflow obstruction (Fahy & Dickey, 2010). Small airways have been recognized as a predominant site of airflow obstruction due to mucus plugging in patients with chronic lung diseases (Van Brabandt et al., 1983; Yanai et al., 1992).

Airway mucus is an extracellular gel in which water and mucous glycoproteins (termed mucins) are the major components (Thornton & Sheehan, 2004). Mucins are produced and stored by serous cells in submucosal glands and epithelial goblet cells along the respiratory tract. These cells secrete mucins constitutively (Rose & Voynow, 2006). However, a number of mechanisms may be responsible for augmenting secretion in response to airway insults, including autonomic efferent innervation, airway intrinsic neurons, and axon reflexes from C‐fiber afferents [reviewed by Rogers (Rogers, (2001))].

Substance P is a tachykinin localized to sensory fibers (C‐fibers) innervating the airway epithelium, glands, blood vessels, and smooth muscle (Chuaychoo et al., 2005; Lundberg et al., 1984). It is also found in airway intrinsic neurons (Dey et al., 1988). Substance P activates G protein‐coupled tachykinin NK1 (SP‐preferring) receptors expressed in submucosal glands and airway epithelial cells (Bai et al., 1995; Mapp et al., 2000). Release of SP and activation of its putative receptors also cause airway smooth muscle constriction in humans (Frossard & Barnes, 1991; Fuller et al., 1987; Naline et al., 1989).

There has been a long‐standing interest in tachykinins as contributors to airway diseases that are characterized by excess mucus. For example, tachykininergic pathways are upregulated in asthma (Cardell et al., 1994; De Vries et al., 2006; Nieber et al., 1992; Ollerenshaw et al., 1991) and COPD (Tian et al., 2000). In experimental animal models of allergic asthma, concentrations of SP in the bronchoalveolar lavage fluid are associated with increased mRNA for the major mucin glycoprotein mucin 5AC (*muc5AC*; Reznikov et al., 2016). Moreover, it was recently demonstrated that vagal sensory neuron‐mediated mucous secretion in the inflamed mouse trachea depends upon SP (Talbot et al., 2020). However, the cellular pathways and mechanism(s) connecting SP to mucous secretion are not well elucidated. Further, whether overexpression of SP without pre‐existing inflammation is sufficient to induce changes in mucous properties is unknown.

In the present study, we tested the hypothesis that AAV‐mediated overexpression of SP in small airways increases mucin secretion ex vivo. Second, we tested the hypothesis that blockade of the nuclear factor kappa‐B (NF‐kB) inducible transcription factors would mitigate SP‐induced changes in mucin secretion (Ni et al., 2016). We studied swine, which exhibit airway anatomy and physiology that closely parallels human airway anatomy and physiology (Judge et al., 2014). Because the study of small airway function in vivo imposes experimental challenges, measurement of airway mucous secretion and transport was assessed in porcine precision‐cut lung slices (pPCLS) ex vivo. Finally, we used porcine airway epithelial cells cultured at the air‐liquid interface to pharmacologically validate the findings.

## 2 MATERIALS AND METHODS

### 2.1 Chemicals and drugs

Acetyl‐beta‐methacholine‐chloride (Sigma‐Aldrich) was dissolved in 0.9% saline for ex vivo application. Bay 11‐7082 (Cat. 196871; Sigma‐Aldrich) was diluted in DMSO and used at a final concentration of 10 µM (Kudo et al., 2012). Substance P (Sigma‐Aldrich) was dissolved in DMSO and used at a final concentration of 100 nM. Final concentration of DMSO (vehicle controls) was 0.1% (vol/vol).

### 2.2 Animals

All protocols and procedures involving animals were carried out in accordance with federal guidelines and approved by the Animal Care and Use Committee at the University of Florida. Lungs were obtained from 24 2–3 days old piglets (Yorkshire‐Landrace) post euthanasia. Data were collected from eight separate cohorts of piglets across approximately 9 months. Animals were sedated with an intramuscular administration of ketamine (20 mg/kg) and xylazine (2 mg/kg), and intravenous propofol (0.8–1.6 mg/kg), followed by euthanasia with intravenous pentobarbital sodium and phenytoin sodium solution (90 mg/kg; EUTHASOL, Henry Schein Animal Health). Immediately after euthanasia, the chest cavity was exposed, and lungs were excised along with trachea and heart. Tissues were kept at 4°C until further processing. On average, the length of storage in 4°C ranged from 3 to 4 h.

### 2.3 Cloning of Sus scrofa TAC1

Swine Substance P was amplified from porcine nucleus tractus solitarius extracted and transcribed mRNA, using the following primers that included NotI sites: Forward 5′‐GCGGCCGCATCGAACATGAAAATCCTCGTG‐3′ and Reverse 5′‐GCGGCCGCAGGTAGTTTATTTACGTCTTCTTTCATAATT‐3′, and High Fidelity Platinum Taq DNA polymerase (Cat. 1304‐011, Invitrogen) and a ProFlex PCR system. The amplification program was as follows: initial denaturation at 94°C for 1 minute, followed by 35 cycles of denaturation at 94°C for 30 s, annealing at 55°C for 35 s, and extension at 68°C for 1 min and 30 s. The amplified DNA was run on 1% agarose gel and extracted from the gel using a QIAquick Gel Extraction kit (Cat. 28704, Qiagen). The extracted DNA was subcloned into a pCR 4‐TOPO vector (Cat. K457502, Invitrogen) and subsequently cloned into the previously described AAV helper vector SC_smCBA_hGFP (Kong et al., 2010) containing a chicken β‐actin (CBA) promoter and a green fluorescent protein (GFP; kindly provided by Dr. Boye from the UF Powell Gene Therapy Center vector core), where the GFP sequence was substituted for the Substance P sequence using NotI enzyme digestion (Cat. R3189S, New England BioLabs) and ligation (Express Link T4 DNA ligase; Cat. A13726, Invitrogen) according to the manufacturer instructions. The helper vector was amplified in One Shot Stabl3 Chemically competent *E*. *coli* (Cat. C7373‐03, Invitrogen) and purified using EndoFree Plasmid Maxi Prep kit (Cat. 12362, Qiagen). Amplified DNA and subsequent insertions were sequenced (GENEWIZ Inc., South Plainfield, NJ) and verified against the predicted *Sus scrofa* tachykinin precursor 1 (*TAC1*), transcript variant X1, mRNA (NCBI Reference Sequence: XM_003130164.6). The proper orientation of insertion was verified using sequencing with the following primer: 5′‐TATGGACATGGCCAGATCTCTCAT‐3′.

### 2.4 Viral vector production

Recombinant AAV vector serotype 2.1 expressing the *Sus scrofa* TAC1 cDNA and the corresponding SC_smCBA_hGFP control vector were produced at the Vector Core Laboratory of the University of Florida (Gainesville, FL).

### 2.5 Porcine precision‐cut lung slices preparation and culture

pPCLS were made as previously described (Reznikov, Meyerholz, Abou Alaiwa, et al., 2018). Briefly, the porpoise lobe was dissected, cannulated, and insufflated with 2% (weight/vol) low‐melting‐point agarose (Invitrogen) in the culture medium. The culture medium consisted of DMEM (Gibco, Thermo Fisher, Grand Island, NY) supplemented with 10% FBS (Gibco, Thermo Fisher), and 1% penicillin/streptomycin. The lobes were kept at 4°C for a minimum period of 30 min that enabled the agarose to polymerize. Once the agarose set, the intermediate third of the lobe was transversally sectioned, and cores of approximately 6 mm diameter were made. The cores were fixed to a pistol using super glue and embedded in 2% (weight/vol) high‐melting‐point agarose (Fisher, Geel, Belgium) in the culture medium. Samples containing a central airway by visual inspection were sliced at a thickness of 300 µm using a vibratome (Compresstome VF‐300‐0Z; Precisionary Instruments) and collected in a reservoir containing DMEM supplemented with 1% penicillin/streptomycin. Lung slices were transferred sequentially into 12‐well culture plates (1 slice per well) containing 2 ml of pre‐warmed culture medium and cultured at 37°C in a humidified atmosphere of 5% CO_2_.

### 2.6 AAV transfection

After approximately 3 h of acclimatation in culture, pPCLS were visualized under an inverted microscope (Olympus, Tokyo, Japan) and only lung slices presenting a central airway with active cilia beating remained in the experiment. pPCLS were transduced with 5 µl of 1.23E13 viral genomes/ml of AAV2.1‐GFP (control) or 5 µl of 1.16E13 AAV2.1‐SP. Forty‐eight hours following transduction, lung slices were snap‐frozen for RNA isolation, fixed for immunostaining, or subjected to ex vivo assays as described below. Samples for RNA isolation were kept at –80°C until further processing. In the first experiment, we treated four lung slices with AAV2.1‐GFP and four lung slices with AAV2.1‐SP. Each pair of lung slices were designated for a single experiment (e.g., immunofluorescence of MUC5AC, immunofluorescence of MUC5B, qRT‐PCR, and mucociliary transport). In the second experiment, we treated three lung slices with AAV2.1‐GFP+vehicle, three lung slices with AAV2.1‐GFP+BAY, three lung slices with AAV2.1‐SP+vehicle, and three lung slices with AAV2.1‐SP+BAY. Lung slices from all four conditions were designated for a single experiment (e.g., immunofluorescence of MUC5AC, immunofluorescence of MUC5B, and qRT‐PCR).

### 2.7 Porcine epithelial airway cells

Airway cells were isolated and cultured at an air‐liquid interface as previously described (Kuan et al., 2019; Reznikov et al., 2019). Cultures were treated with 100 nM SP (Sigma Aldrich) for two consecutive days to mimic the 48‐h exposure in lung slices. The dose was selected based upon a pilot study that suggested that of the three concentrations tested, 100 nM was the lowest that increased MUC5AC.

### 2.8 Ex vivo mucus transport and airway constriction assays

Mucociliary transport was measured using methods similar to those described for trachea (Hoegger et al., 2014; Liao, Kuan, et al., 2020) with minor modifications. Briefly, pPCLS were transferred into dental wax‐coated 60 mm Petri dishes and submerged in 5 ml of prewarmed PBS (with Ca^2+^ and Mg^2+^, at pH 7.4) containing 10 mM HEPES, 100 μM bumetanide and red fluorescent nanospheres (FluoSpheres carboxylate‐modified microspheres, 0.04 µm; Invitrogen; 1:1000 dilution). Samples were placed onto a heated stage at 37°C and visualized under a fluorescence microscope Zeiss Axio Zoom.V16 (Carl Zeiss). Images were acquired every 1 minute for 35 minutes. After 5 min of baseline measurements, methacholine was administered directly into the incubation solution at a dose of 0.004 mg/ml (Liao, Kuan, et al., 2020). Mucociliary transport was assessed for 30 min after methacholine stimulation by real‐time visualization of the nanospheres that attached to mucus. To measure the movement of fluorescently labeled mucus, we utilized IMARIS computer‐assigned particle‐tracking that uses validated algorithms (Jaqaman et al., 2008). In addition to the mucociliary transport, we evaluated airway constriction in response to methacholine stimulation. Using ImageJ (FIJI version 1.52a, National Institute of Health), we measured the airway luminal area at minute 5 (right before methacholine addition), and at minute 10 (5 min after methacholine addition) and calculated the percentage of area constricted. One pair of pPCLS where airways appeared to be precontracted (i.e., closed lumen upon microscopic examination) was excluded from the analysis.

### 2.9 Drug treatment of lung slices

We aimed to test whether blockade of the NF‐kβ pathway mitigates SP overexpression‐mediated effects on MUC5AC. pPCLS were prepared, cultured, and transduced with AAV as detailed above. Twenty hours after transduction with AAV2.1‐GFP or ‐SP, lung slices were treated with the irreversible NF‐kβ inhibitor Bay 11‐7082 (Sigma‐Aldrich) at a final concentration of 10 µM (IC50 ≈ 10 µM; Kudo et al., 2012) or 0.1% (vol/vol) DMSO as vehicle control. After 2 h of incubation, the culture medium was replaced by a fresh medium (Busse et al., 2009). Lung slices were cultured for another 26 h to mirror the time course of our original observation and studies and thereafter snap‐frozen for posterior RNA isolation or fixed for immunostaining. Spent culture medium was also collected for ELISA analyses. Spent media and samples for RNA isolation were kept at –80°C until assayed.

### 2.10 RNA isolation and cDNA synthesis

Total RNA was isolated using QIAzol Lysis Reagent (Qiagen) and the RNeasy Lipid Tissue Mini Kit (Qiagen) as per the manufacturer's instructions. All samples were subjected to on‐column DNase (Qiagen) digestion. RNA concentrations was assessed using a NanoDrop spectrophotometer (Thermo Fisher). Total RNA (150 ng) was reverse transcribed using SuperScript IV VILO Master Mix (Invitrogen) according to the manufacturer's instructions.

### 2.11 Pig inflammatory cytokines and receptors PCR array

Abundance of 81 transcripts related to inflammatory pathways were assessed using porcine RT2 Profiler PCR Arrays (PASS‐011ZF, Qiagen) as previously described (Reznikov et al., 2019; Reznikov, Meyerholz, Kuan, et al., 2018). Reactions were carried out in a final volume of 25 µL in 96‐well plates using fast SYBR green master mix (Applied Biosystems) according to the manufacturer's instructions. The program consisted of an initial denaturation step at 95°C for 10 min, followed by 50 cycles each of 10 s at 95°C, annealing at 60°C for 10 s, and extension at 72°C for 10 s. Melting curves were plotted by stepwise increases in the temperature from 65 to 97°C. Relative abundances were calculated using the 2–ΔΔCt method (Livak & Schmittgen, 2001) after the normalization of the Cq values by the geometric mean of the endogenous controls Cq values. *ACTG1*, *B2M*, *GAPDH*, and *RPL13A* served as endogenous controls. Numbers of samples needed to detect a statically significant difference in the inflammatory‐directed PCR arrays were based upon our previous studies (Reznikov et al., 2019; Reznikov, Meyerholz, Kuan, et al., 2018).

### 2.12 Immunofluorescence

pPCLS or airway epithelial cell cultures were fixed in 2% paraformaldehyde for 30 min and kept immersed in PBS (Ca^2+^ and Mg^2+^ free) at 4°C. Samples were immunostained as previously described (Liao, Kuan, et al., 2020) with minor modifications. Briefly, lung slices were permeabilized 0.15% (vol/vol) Triton X‐100 in PBS for 30 min at room temperature. Samples were blocked in SuperBlock™ (Thermo Fisher) added of 4% (vol/vol) normal goat serum for 2 h at room temperature, and then incubated with the primary antibody overnight at room temperature. Lung slices were immunostained with the following antibodies: mouse anti‐MUC5AC (Clone 45M1, Cat. MA512178, Thermo Fisher; 1:1000 dilution), and rabbit anti‐MUC5B (Cat. 20119, Santa Cruz; or Cat. HPA008246, Sigma‐Aldrich; 1:1000 dilution). After washing steps, pPCLS were incubated with goat anti‐rabbit or goat anti‐mouse secondary antibodies conjugated to Alexa‐Fluor 568 (Thermo Fisher; 1:1000 dilution) for 1 h at room temperature. Nuclei were counter‐stained with Hoechst 33342 (Thermo Fisher) for 10 min. Thereafter, pPCLSs were mounted onto Superfrost Plus microscope slides (Thermo Fisher) in 90% glycerol and imaged with Zeiss Axio Zoom.V16 microscope equipped with ZenPro software (Carl Zeiss) using identical acquisition settings across samples. Signal intensity in the airway mucosa was quantified using the polygon selection tool in ImageJ. Background fluorescence was subtracted manually as described (Liao, Collins, et al., 2020). For airway cultures, two image fields were captured and mean signal intensity reported using Zeiss ZenPro software. The analyses were carried out by two to three investigators, and the average values were reported.

### 2.13 Enzyme‐linked immunosorbent assay (ELISA) for Substance P and interleukin 10

Porcine SP and IL10 in spent media were detected using, respectively, a competitive substance P ELISA Kit (Cat. ab133029, Abcam) and a solid phase sandwich IL10 ELISA kit (Cat. P1000, R&D Systems). All procedures were carried out according to the manufacturer's protocols. Samples were assayed in duplicates and read using a filter‐based accuSkan FC microphotometer (Fisher Scientific) at 405 nm. For the SP ELISA, the 7‐point standard curve was diluted in culture medium and ranged from 0 to 10,000 pg/mL. Concentrations were determined from the standards plotted in a 4‐parameter logistic sigmoidal curve (*R* ^2^ > 0.99). The intra‐ and inter‐assay coefficients of variability were, respectively, 6.7% and 4.2%. For the IL10 ELISA, concentrations were determined from the standards plotted in a linear curve (*R* ^2^ > 0.98). The intra‐ and inter‐assay coefficients of variability were, respectively, 2.6%–4.2% and 4.5%–7.2%.

### 2.14 Data analysis

All analyses and visualization were performed using GraphPad Prism 8.3 (GraphPad Software). We used a two‐tailed paired T‐test to examine the effects of overexpression of SP on outcome measures since treatments were applied in sequential lung slices within a single individual. In our second set of experiments, our major hypothesis was that NF‐kβ prevented the effects of SP overexpression. Therefore, we calculated the delta for outcome measures relative to their respective controls and used a two‐tailed paired T‐test, again to account for the sequential matching of lung slice treatments within animals. Inflammatory‐directed PCR arrays data were analyzed by paired T‐test. We initially tested for sex differences, but none were detected and therefore the data were combined. Statistical significance was determined as *p* ≤ 0.05 and a probability of 0.05 < *p* ≤  0.10 indicated a trend toward significance.

## 3 RESULTS

### 3.1 AAV‐mediated overexpression of Substance P in porcine precision‐cut lung slices increases MUC5AC

**FIGURE 1:**
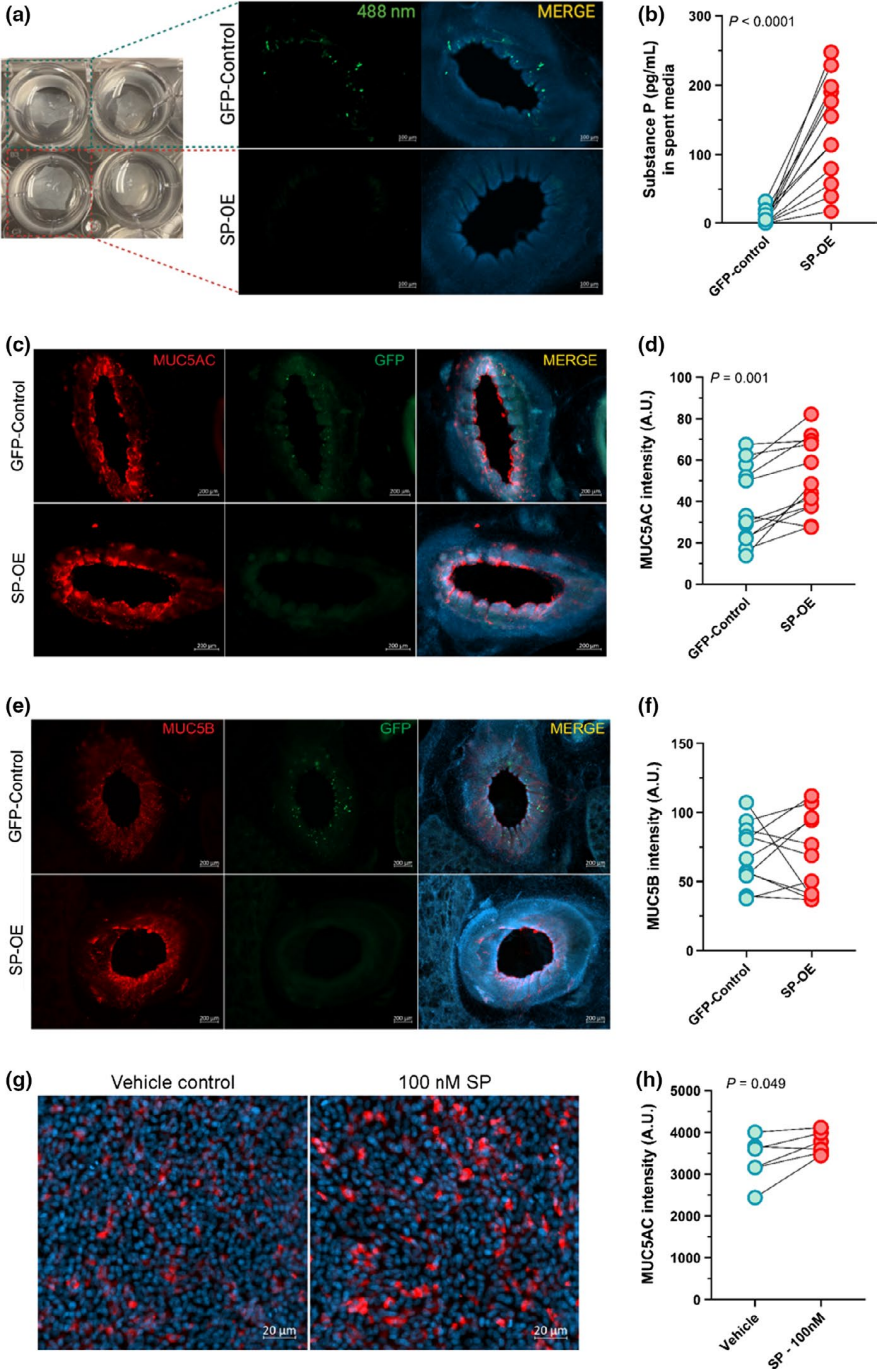
Overexpression of Substance P increases MUC5AC. (a) Overview of the experimental model. (b) Concentrations of SP in lung slice culture media 48 h post‐AAV transduction (*n* = 12; 6 males, 6 females). (c) Antibody‐labeling of mucin 5AC (MUC5AC; red) and (d) MUC5AC signal intensity quantification (*n* = 12; 6 males, 6 females). (e) Antibody‐labeling of mucin 5B (MUC5B; red) and (f) MUC5B signal intensity quantification (*n* = 11; 6 females, 5 males). (g) MUC5AC protein (red) in vehicle and SP‐treated cultured swine airway epithelia and (h) quantification of MUC5AC fluorescence intensity (*n* = 6 individual cultures from six unique piglets; three females, three males). Nuclei are shown in blue (Hoechst). Groups were compared by paired *T*‐test. Data are shown as arbitrary units (A.U.). AAV, Adeno‐associated virus; GFP, green fluorescent protein; OE, overexpression; SP, Substance P. Lines show matched pairs. Mean, standard error of the mean (SEM), Log2 Fold‐change, and *p*‐values are shown in Tables S1–S2.

We first confirmed overexpression of SP by measuring the concentration of SP in lung slice media 48 h after transduction with AAV. We found a marked increase of SP protein in all samples transduced with AAV carrying the *TAC1* gene compared to GFP‐controls (*p* < 0.0001; Figure 1B). Substance P concentration averaged 7.6 and 135.0 pg/ml in culture media from GFP‐control and SP‐overexpressed lung slices, respectively.

We next assessed airway mucin secretion by measuring the major secretory gel‐forming mucins MUC5AC and MUC5B using antibody labeling. We found a significant increase in the signal intensity of MUC5AC in the central airway of lung slices overexpressing SP (*p* = 0.0015; Figure 1c,d; Table S1). No difference in MUC5B signal intensity was observed (Figure 1e,f). Using porcine airway epithelia cultured at the air‐liquid interface, we confirmed that treatment with exogenous SP augmented MUC5AC expression (Figure 1g,h; Table S2).

### 3.2 Substance P overexpression tends to decrease mucociliary transport but does not alter contractile properties in methacholine‐challenged small airways ex vivo

**FIGURE 2:**
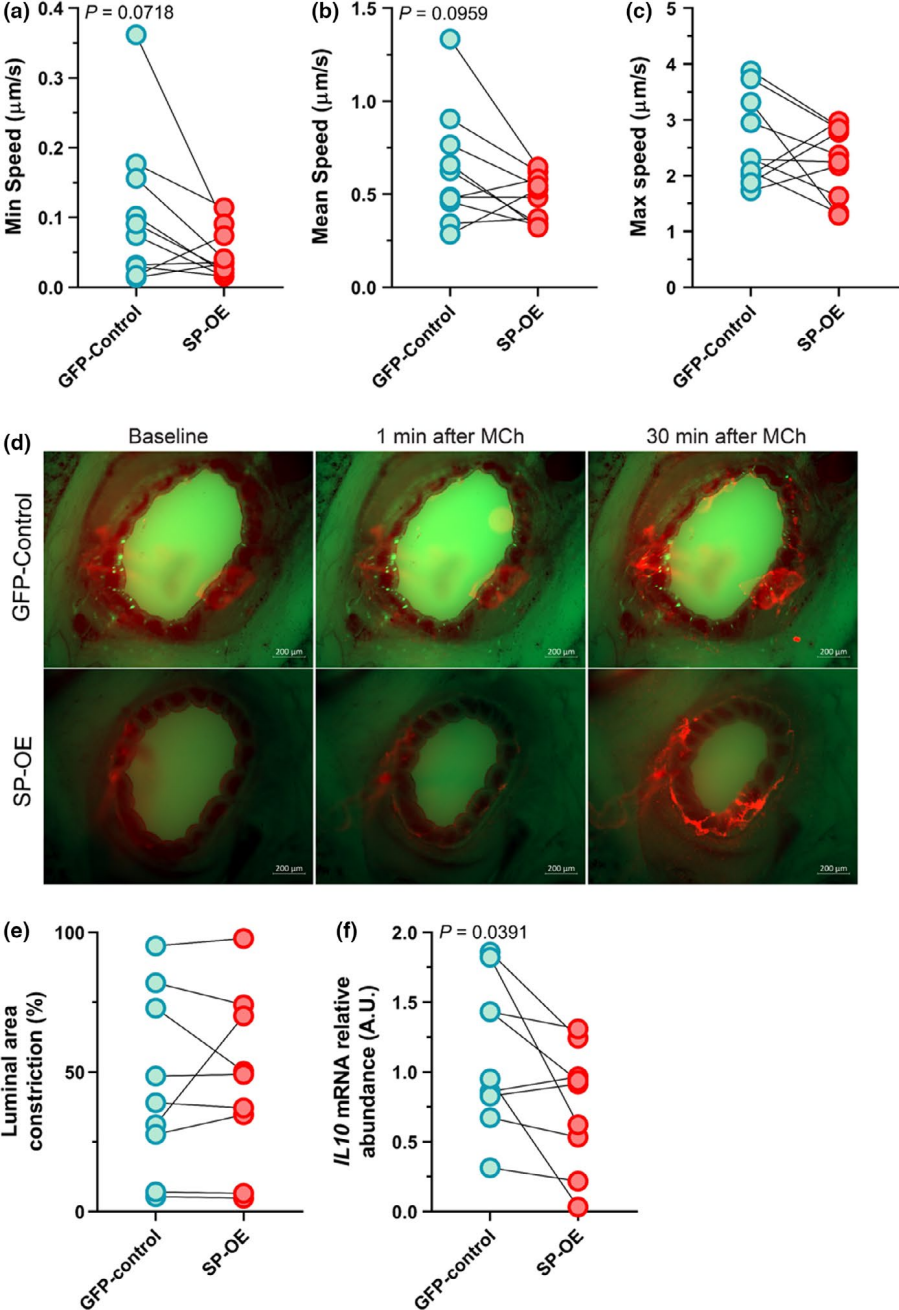
Overexpression of Substance P decreases IL10. (a) Minimum, (b) mean, and (c) maximum mucociliary transport speeds (*n* = 10; 5 females, 5 males). (d) Representative images of baseline, 1 min after methacholine (MCh) and 30 min after MCh (at the conclusion of the experiment) in lung slices. Fluorescent red is mucus labeled with beads. (e) Percentage of luminal constriction from baseline (*n* = 9; 5 females, 4 males). (f) Relative abundance of transcripts for Interleukin 10 (*IL10)* in GFP‐control or SP‐OE lung slices as determined by qRT‐PCR (*n* = 9; 5 females, 4 males). Data are shown as arbitrary units (A.U.). Groups were compared by paired *T*‐test. AAV, Adeno‐associated virus; GFP, green fluorescent protein; OE, overexpression; SP, Substance P. Lines show matched pairs. Mean, standard error of the mean (SEM), Log2 Fold‐change, and P‐values are shown in Table S1–S3.

Abnormal and/or excess mucus often results in impaired mucociliary transport (Liao, Kuan, et al., 2020). We found that overexpression of SP tended to decrease minimum and mean transport speeds of fluorescently labeled mucus in the central airway of precision‐cut lung slices compared to the GFP‐controls (*p* = 0.072 and 0.096, respectively; Figure 2a,b; Table S1). Maximum speeds were unaffected (Figure 2c). Central airway contraction in response to methacholine stimulation was also measured, but no significant differences between groups were detected (Figure 2d,e). These data suggest that overexpression of SP leads to a mild impairment in mucociliary transport. However, under the same conditions, airway contractile properties were unaffected.

### 3.3 Substance P overexpression in lung slices decreases interleukin 10 transcript abundance

Substance P has pro‐inflammatory properties, and it is well accepted that inflammation increases MUC5AC expression in the airway. Thus, we examined inflammation using inflammatory‐directed quantitative real‐time PCR arrays. Evaluation of 81 transcripts revealed that mRNA abundance for *IL10* was mildly decreased (1.5‐fold, *p* = 0.04) in lung slices where SP was overexpressed compared to GFP‐controls (Figure 2f, Table S3). No other changes were detected. These data suggest that overexpression of SP selectively decreases *IL10* mRNA abundance.

### 3.4 Substance P‐mediated increases in MUC5AC are mitigated by NF‐kβ inhibition

**FIGURE 3:**
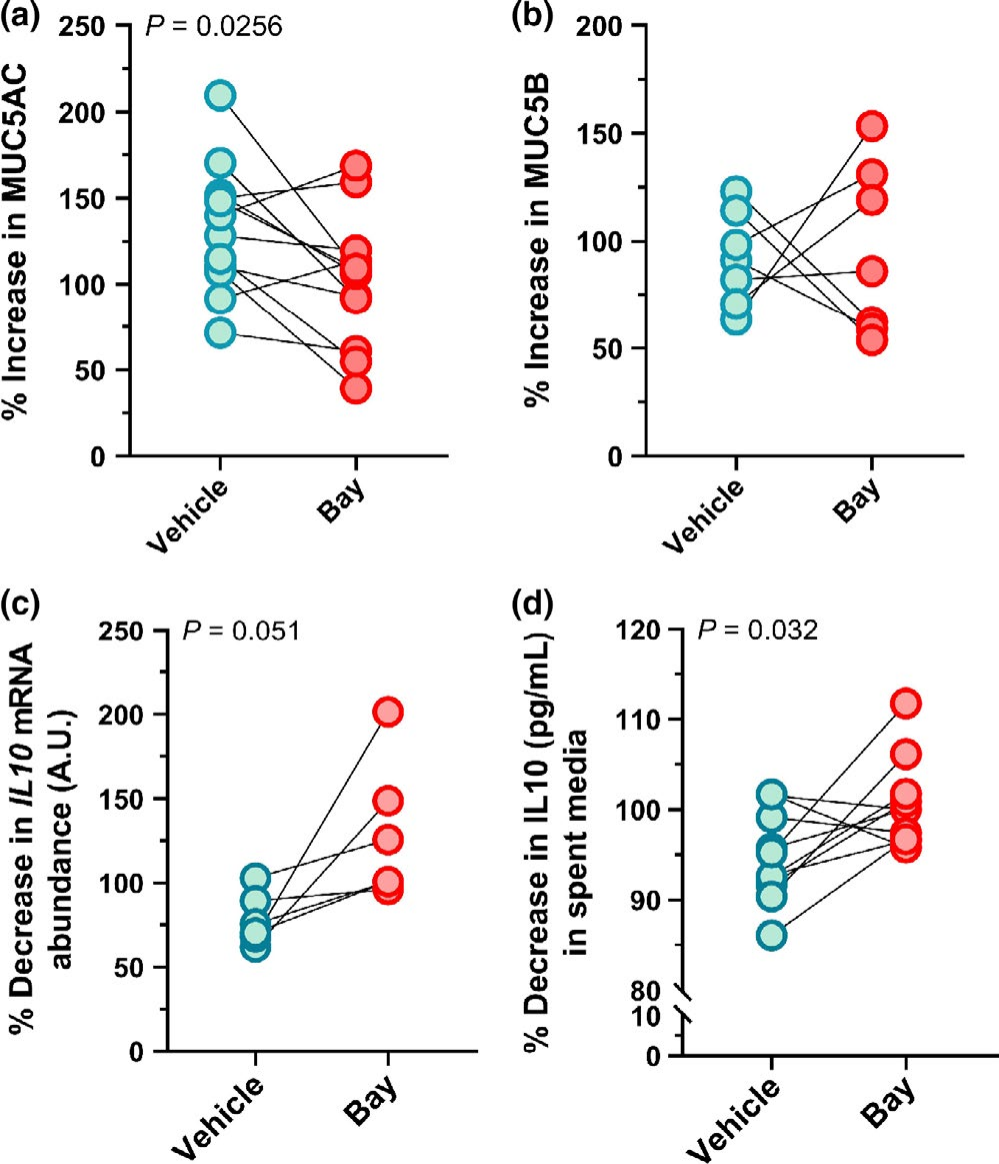
NF‐kβ inhibition mitigates the effects of Substance P overexpression on MUC5AC and IL10. (a) Quantification of % increase in MUC5AC (*n* = 12; 6 females, 6 males) and (b) % increase in MUC5B (*n* = 7; 4 females, 3 males) relative to respective controls. (c) Quantification of % decrease in Interleukin 10 (*IL10)* mRNA (*n* = 6; 3 females, 3 males) and (d) IL10 protein (*n* = 10; 5 females, 5 males). Data are the normalized effects of overexpression of SP compared to GFP controls (e.g., % of GFP control) in the presence of vehicle or BAY 11‐7082. Data are evaluated with a paired T‐test. GFP, green fluorescent protein; SP, Substance P; Bay, BAY 11‐7082. Mean, standard error of the mean (SEM), and *p*‐values of the raw data are shown in Table S4.

Because SP induces NF‐kβ pathway activation (Sun et al., 2008) and NF‐kβ activation increases MUC5AC (Fujisawa et al., 2009; Kraft et al., 2008), we tested the hypothesis that SP‐mediated induction of MUC5AC requires the inducible NF‐kβ transcription factor complex. Similar to our original findings, overexpression of SP increased MUC5AC signal intensity in the central airways compared to GFP‐control (Figure 3a). Pharmacologic inhibition of NF‐kβ with the drug BAY 11‐7082 (Pierce et al., 1997) mitigated this effect (Figure 3a; Table S4). MUC5B expression was unaffected in response to NF‐kβ inhibitions (Figure 3b; Table S4). Lastly, to determine whether SP‐mediated reductions in *IL10* mRNA might also involve NF‐kβ‐dependent mechanisms, we analyzed IL10 mRNA and protein abundance with and without NF‐kβ inhibition. Blockade of NF‐kB mitigated the effects caused by overexpression of SP on *IL10* mRNA in lung slices (*p* = 0.051) and IL10 protein in culture media (*p* = 0.032; Figure 3c,d). These data suggest that NF‐kβ pathways are involved in SP‐mediated induction of MUC5AC and inhibition of IL10.

## 4 DISCUSSION

Mucus overproduction and hypersecretion are prominent pathological findings in chronic respiratory diseases, such as asthma (Dey et al., 1988). The tachykinin SP has been implicated as a key driver of abnormal mucous secretion in asthma (Borish et al., 1996; Evans et al., 2015). However, the mechanism by which SP engenders abnormal mucous secretion and the extent to which inflammation is required has remained unknown. Our data suggest that the overexpression of SP increases MUC5AC through a pathway involving NF‐kβ. Overexpression of SP also decreased IL10, which can be produced by the epithelium (Fahy & Dickey, 2010) and is considered an inhibitory cytokine of airway inflammation (Frossard & Barnes, 1991). Thus, these data further highlight SP as a key driver of abnormal mucus secretion and underscore NF‐kβ signaling as an important mediator.

MUC5AC and MUC5B are the major secreted gel‐forming mucins in the respiratory epithelium (Kirkham et al., 2002; Thornton et al., 2008). In the mouse, MUC5AC contributes to airway hyperreactivity (Evans et al., 2015) and mucus plugging, whereas MUC5B is required for airway homeostasis, mucociliary clearance, and anti‐bacterial defenses (Roy et al., 2014). In the pig, elimination of airway submucosal glands, which predominantly secrete MUC5B, also impairs mucociliary clearance and airway host defenses (Ostedgaard et al., 2020). We found that overexpression of SP selectively increased MUC5AC. This finding is in agreement with other studies reporting a positive correlation between MUC5AC and neurokinin A (NKA) protein expression in the sputum of asthmatics (Hallstrand et al., 2007). Although our studies do not explain why overexpression of SP increased MUC5AC and not MUC5B, it is possible that expression of the NK1 receptor is enhanced in porcine goblet cells. Support for this statement is provided by DeSwert and colleagues who determined that mice lacking NK1 receptor exhibited less goblet cell hyperplasia in response to allergen sensitization compared to wild‐type controls (De Swert et al., 2004). It also possible that the regulation of MUC5B in the small airways does not rely upon NF‐kβ signaling but rather other elements, such as AP‐1 or Sp1 (Thai et al., 2008).

We found that SP overexpression also decreased *IL10* transcript abundance. IL10 is produced by several cells of the immune system in the respiratory tract (McGuirk et al., 2002; Sun et al., 2009), but has also been shown to be produced by the epithelium (Bonfield et al., 1995). IL10 can act as an inhibitory cytokine of airway inflammation, as its constitutive expression in the respiratory tract maintains tolerance to allergens and aerosols (Tournoy et al., 2000). In asthmatic (Borish et al., 1996), CF (Bonfield et al., 1995) and COPD (Takanashi et al., 1999) patients, decreased IL10 production has been reported. Although we do not know the consequences of reduced IL10 in the absence of inflammation in the airway, targeted disruption of IL10 in mice produces spontaneous enterocolitis (Kuhn et al., 1993). In our study, the mechanism responsible for decreased IL10 in response to overexpression SP is unclear, although blockage of the NF‐kβ signaling cascade mitigated the effects of SP on IL10 mRNA and protein, suggesting its involvement. Interestingly, *IL10 *has been assigned as an NF‐kβ target gene (Yang et al., 2016), however, whether the transcription factor complex induces or inhibits *IL10* transcription is not clear. Previous work suggests that in dendritic cells, NK1 receptor activation decreases IL10 synthesis and secretion primarily through a CREB/TORC2 signaling pathway (Janelsins et al., 2013). Thus, our data are consistent with a role for SP in regulating IL10 and shed new light on responsible mechanisms.

We overexpressed the *TAC1* gene, which gives rise to multiple tachykinins products [reviewed here (Atanasova & Reznikov, 2018)]. While the AAV‐2.1 serotype used in this study has a natural tropism for neurons (Haggerty et al., 2020), other cells are likely to be transduced (Yan et al., 2013). Therefore, the *TAC1* gene will be under cell‐type specific regulation. Thus, it is possible that other tachykinins contributed to the observed increase in MUC5AC. To address this, further studies using tachykinins antagonists could be employed. However, since prior work suggests that only activity of SP on NK1 receptors in the porcine trachea can cause direct activation of mucus‐producing cells (Phillips et al., 2003), such studies may be less informative.

Our study has strengths and limitations. Major strengths include the use of porcine airways, which closely resemble those of humans (Judge et al., 2014), and the use of AAV2.1 to overexpress SP in the absence of pre‐existing inflammation. For example, although many previous studies have shown that SP increases mucous secretion acutely using physiologically relevant concentrations, our strategy to overexpress SP using AAV2.1 allowed for the isolation of the sole effects of overexpression of SP on airway mucin secretion and production in the absence of inflammation. This is in contrast to prior work that has focused primarily on SP on a background of inflammation. Moreover, because the effect of overexpression of SP on MUC5AC was blocked by NF‐kβ, our findings suggest that SP is not merely causing mucous secretion but increasing mucous production. This is important because increased production of MUC5AC has not previously been directly attributed to SP without pre‐existing inflammation. Additionally, by using AAV‐mediated overexpression, it is expected that a more sustained production and release of SP over time occurs compared to the addition of exogenous SP. This is of special importance since SP has a short half‐life in tissues (i.e., ranging from seconds to tens of minutes) (McGregor & Bloom, 1983). Therefore, exogenous application of SP might not fully mimic what occurs in disease states. Our study also presents limitations. We performed studies in vitro as opposed to in vivo. Although this approach allowed for the isolation of the effects of overexpression of SP on the airway only, it is possible that the immune system and nervous system modify the responses to overexpression of SP in vivo. Additionally, our studies were performed on a relatively short time scale (48 h), and therefore we do not know the extent to which SP‐mediated phenotypes remain long‐term. This may be an important consideration in the context of airway diseases, which are typically chronic. Therefore, time course studies are needed.

In summary, our data highlight SP as a key molecule that selectively increases MUC5AC in the absence of overt inflammation. The increase in MUC5AC appears to be mediated through NF‐kβ signaling. Thus, these data suggest that both inhibitions of SP signaling and blockade of the NF‐kβ pathway may be potential points of therapeutic intervention for chronic airway diseases, such as asthma.

## CLASSIFICATION

Biological Sciences; Physiology.

## CONFLICT OF INTEREST

Authors declare no competing interests.

## AUTHOR CONTRIBUTIONS

MS, KRA, and LRR participated in the conception and design of the research. MS, YSL, ENC, VS, LB, and LRR performed the experiments. MS and LRR analyzed the data. MS and LRR interpreted the results of the experiments. MS and LRR prepared the figures. MS drafted the manuscript. All authors edited and reviewed the manuscript.

## Supporting information



Table S1‐S4
