# Supplementary material for: Overexpression of Substance P in pig airways increases MUC5AC through an NF‐kβ pathway
Source: Physiol Rep. 2021 Feb 13;9(3):e14749. doi: 10.14814/phy2.14749 (PMC7881348; doi:10.14814/phy2.14749)
Supplement: Supplementary file 1 — Table S1‐S4 [file PHY2-9-e14749-s001.docx]

**Supplemental Table S1.** Mean, standard error of the mean (SEM), Log2 Fold-change and P-values for all features measured in lung slices overexpressing GFP (GFP-control) or Substance P (SP-OE).

| Outcome | GFP-control (Mean ± SEM) | SP-OE (Mean ± SEM) | Log2 Fold-change | P-value |
| --- | --- | --- | --- | --- |
| Substance P (pg/mL) in spent media | 7.595 ± 3.243 | 134.975 ± 21.939 | 4.151 | <0.0001 |
| MUC5AC intensity in airways (arbitrary unit) | 38.163 ± 5.414 | 51.278 ± 5.274 | 0.426 | 0.001 |
| MUC5B intensity in airways (arbitrary unit) | 69.398 ± 6.826 | 69.060 ± 8.951 | −0.007 | 0.972 |
| Min speed (µm/s) | 0.105 ± 0.034 | 0.047 ± 0.011 | −1.160 | 0.072 |
| Mean speed (µm/s) | 0.634 ± 0.098 | 0.478 ± 0.040 | −0.407 | 0.096 |
| Max speed (µm/s) | 2.617 ± 0.250 | 2.252 ± 0.203 | −0.217 | 0.247 |
| Luminal area constriction (%) | 45.449 ± 10.678 | 47.210 ± 10.230 | 0.055 | 0.754 |

**Supplemental Table S2.** Mean, standard error of the mean (SEM), Log2 Fold-change and P-value for MUC5AC intensity measured in cultured swine airway epithelial cells treated with vehicle or Substance P (SP; 100nM).

| Outcome | Vehicle (Mean ± SEM) | SP (Mean ± SEM) | Log2 Fold-change | P-value |
| --- | --- | --- | --- | --- |
| MUC5AC intensity (arbitrary unit) | 3338.548 ± 222.954 | 3737.414 ± 109.631 | 0.163 | 0.049 |

**Supplemental Table S3.** List of 81 transcripts queried through Pig Inflammatory Cytokines and Receptors PCR arrays in lung slices overexpressing GFP (GFP-control) or Substance P (SP-OE).

| Gene name | GFP-Control (Mean ± SEM) | SP-OE (Mean ± SEM) | Log2 Fold-change | P-value |
| --- | --- | --- | --- | --- |
| *AIMP1* | 1.024 ± 0.077 | 1.040 ± 0.061 | 0.022 | 0.843 |
| *BMP2* | 1.258 ± 0.283 | 1.089 ± 0.283 | −0.207 | 0.263 |
| *C5* | 1.481 ± 0.405 | 1.456 ± 0.382 | −0.025 | 0.869 |
| *CCL1* | 1.488 ± 0.601 | 1.075 ± 0.171 | −0.469 | 0.439 |
| *CCL17* | 1.265 ± 0.271 | 1.369 ± 0.411 | 0.114 | 0.823 |
| *CCL2* | 1.009 ± 0.047 | 1.056 ± 0.123 | 0.067 | 0.695 |
| *CCL20* | 1.397 ± 0.371 | 1.292 ± 0.281 | −0.113 | 0.496 |
| *CCL21* | 1.241 ± 0.302 | 1.251 ± 0.316 | 0.012 | 0.889 |
| *CCL22* | 1.343 ± 0.450 | 1.228 ± 0.371 | −0.129 | 0.524 |
| *CCL3L1* | 1.251 ± 0.256 | 1.224 ± 0.268 | −0.031 | 0.773 |
| *CCL4* | 1.291 ± 0.271 | 1.296 ± 0.304 | 0.006 | 0.945 |
| *CCL5* | 1.154 ± 0.230 | 1.246 ± 0.268 | 0.111 | 0.582 |
| *CCL8* | 1.179 ± 0.225 | 1.116 ± 0.160 | −0.079 | 0.497 |
| *CCR1* | 1.042 ± 0.102 | 1.043 ± 0.098 | 0.001 | 0.991 |
| *CCR10* | 1.210 ± 0.196 | 1.207 ± 0.299 | −0.004 | 0.991 |
| *CCR2* | 1.265 ± 0.226 | 2.881 ± 0.797 | 1.188 | 0.091 |
| *CCR3* | 1.221 ± 0.217 | 1.320 ± 0.340 | 0.113 | 0.776 |
| *CCR4* | 1.500 ± 0.337 | 1.562 ± 0.310 | 0.058 | 0.865 |
| *CCR5* | 1.124 ± 0.191 | 1.136 ± 0.187 | 0.016 | 0.891 |
| *CCR7* | 1.346 ± 0.371 | 0.993 ± 0.178 | −0.439 | 0.413 |
| *CD40LG* | 3.030 ± 1.050 | 3.300 ± 1.055 | 0.123 | 0.661 |
| *CD70* | 1.061 ± 0.143 | 0.654 ± 0.190 | −0.699 | 0.153 |
| *CSF1* | 1.121 ± 0.180 | 1.113 ± 0.151 | −0.010 | 0.949 |
| *CSF2* | 1.193 ± 0.225 | 1.154 ± 0.221 | −0.047 | 0.721 |
| *CSF3* | 1.404 ± 0.379 | 1.419 ± 0.442 | 0.015 | 0.953 |
| *CXCL10* | 3.231 ± 1.855 | 3.070 ± 1.596 | −0.074 | 0.713 |
| *CXCL11* | 1.283 ± 0.239 | 1.370 ± 0.281 | 0.095 | 0.727 |
| *CXCL12* | 1.113 ± 0.131 | 1.097 ± 0.129 | −0.020 | 0.696 |
| *LOC396594* | 1.188 ± 0.241 | 1.208 ± 0.233 | 0.024 | 0.839 |
| *CXCL9* | 2.159 ± 0.811 | 1.539 ± 0.602 | −0.488 | 0.201 |
| *CXCR2* | 1.079 ± 0.149 | 0.856 ± 0.101 | −0.334 | 0.305 |
| *CXCR4* | 1.400 ± 0.332 | 1.500 ± 0.396 | 0.099 | 0.296 |
| *FASLG* | 1.108 ± 0.154 | 1.557 ± 0.422 | 0.490 | 0.393 |
| *FLT3LG* | 1.122 ± 0.145 | 1.107 ± 0.221 | −0.020 | 0.945 |
| *IFNG* | 2.860 ± 2.034 | 1.753 ± 0.402 | −0.706 | 0.614 |
| ***IL10*** | **1.131 ± 0.177** | **0.754 ± 0.145** | −0.585 | **0.039** |
| *IL10RA* | 1.119 ± 0.172 | 1.305 ± 0.264 | 0.221 | 0.469 |
| *IL10RB* | 1.036 ± 0.101 | 1.084 ± 0.110 | 0.065 | 0.647 |
| *IL12B* | 1.182 ± 0.214 | 0.961 ± 0.258 | −0.298 | 0.325 |
| *IL13* | 1.245 ± 0.227 | 1.666 ± 0.558 | 0.420 | 0.470 |
| *IL16* | 1.062 ± 0.133 | 1.088 ± 0.179 | 0.035 | 0.902 |
| *IL17A* | 1.242 ± 0.362 | 2.097 ± 0.555 | 0.756 | 0.356 |
| *IL17F* | 1.463 ± 0.508 | 1.093 ± 0.352 | −0.420 | 0.607 |
| *IL18* | 1.016 ± 0.067 | 1.167 ± 0.184 | 0.200 | 0.291 |
| *IL18R1* | 1.061 ± 0.138 | 0.923 ± 0.165 | −0.201 | 0.477 |
| *IL1A* | 1.703 ± 0.482 | 1.416 ± 0.485 | −0.266 | 0.269 |
| *IL1B* | 1.512 ± 0.340 | 1.842 ± 0.377 | 0.285 | 0.471 |
| *IL1RN* | 1.170 ± 0.229 | 1.196 ± 0.207 | 0.032 | 0.553 |
| *IL21* | 1.405 ± 0.288 | 2.076 ± 0.760 | 0.563 | 0.467 |
| *IL23A* | 2.403 ± 1.483 | 1.297 ± 0.385 | −0.889 | 0.467 |
| *IL27* | 1.077 ± 0.130 | 1.060 ± 0.242 | −0.022 | 0.957 |
| *IL2RG* | 1.108 ± 0.172 | 1.339 ± 0.283 | 0.272 | 0.472 |
| *IL4* | 1.151 ± 0.184 | 1.393 ± 0.186 | 0.275 | 0.249 |
| *IL4R* | 1.110 ± 0.172 | 1.046 ± 0.212 | −0.085 | 0.673 |
| *IL5* | 1.085 ± 0.142 | 1.092 ± 0.212 | 0.010 | 0.977 |
| *IL5RA* | 1.255 ± 0.271 | 1.087 ± 0.276 | −0.207 | 0.731 |
| *IL6* | 1.050 ± 0.109 | 0.996 ± 0.132 | −0.076 | 0.688 |
| *IL6R* | 1.026 ± 0.078 | 1.091 ± 0.131 | 0.089 | 0.598 |
| *IL6ST* | 1.043 ± 0.104 | 1.027 ± 0.117 | −0.023 | 0.880 |
| *IL7* | 1.035 ± 0.094 | 1.036 ± 0.180 | 0.003 | 0.992 |
| *IL7R* | 1.115 ± 0.198 | 1.017 ± 0.210 | −0.132 | 0.561 |
| *IL9* | 1.429 ± 0.309 | 1.471 ± 0.372 | 0.042 | 0.915 |
| *LIF* | 1.092 ± 0.161 | 0.948 ± 0.252 | −0.204 | 0.357 |
| *OSM* | 1.186 ± 0.257 | 1.217 ± 0.267 | 0.037 | 0.937 |
| *IL17B* | 1.946 ± 0.751 | 1.927 ± 0.742 | −0.014 | 0.956 |
| *IL33* | 1.049 ± 0.116 | 1.093 ± 0.108 | 0.059 | 0.539 |
| *TNFSF14* | 1.271 ± 0.241 | 1.339 ± 0.326 | 0.075 | 0.792 |
| *IL9R* | 1.185 ± 0.183 | 1.249 ± 0.285 | 0.075 | 0.826 |
| *LOC100621682* | 1.201 ± 0.188 | 1.243 ± 0.307 | 0.050 | 0.878 |
| *IL2RB* | 1.526 ± 0.397 | 1.664 ± 0.473 | 0.126 | 0.621 |
| *LTA* | 1.309 ± 0.261 | 1.843 ± 0.447 | 0.494 | 0.239 |
| *LTB* | 1.035 ± 0.095 | 1.317 ± 0.322 | 0.347 | 0.305 |
| *MIF* | 1.016 ± 0.061 | 0.982 ± 0.045 | −0.050 | 0.440 |
| *NAMPT* | 1.024 ± 0.081 | 1.065 ± 0.087 | 0.057 | 0.568 |
| *SPP1* | 1.208 ± 0.229 | 1.211 ± 0.222 | 0.004 | 0.939 |
| *TGFB2* | 1.182 ± 0.252 | 1.176 ± 0.207 | −0.008 | 0.941 |
| *TNF* | 1.421 ± 0.299 | 1.068 ± 0.258 | −0.412 | 0.214 |
| *TNFRSF11B* | 1.165 ± 0.244 | 1.070 ± 0.290 | −0.122 | 0.681 |
| *TNFSF13B* | 1.474 ± 0.493 | 1.158 ± 0.169 | −0.347 | 0.396 |
| *TNFSF4* | 1.158 ± 0.245 | 1.450 ± 0.444 | 0.324 | 0.617 |
| *VEGFA* | 1.068 ± 0.136 | 1.206 ± 0.154 | 0.175 | 0.213 |

**Supplemental Table S4.** Mean, standard error of the mean (SEM), Log2 Fold-change and P-values for all features measured in lung slices overexpressing GFP or Substance P (SP), and treated with vehicle or NF-kβ inhibitor Bay 11-7082 (10 µM).

| Outcome | GFP+vehicle (Mean ± SEM) | SP+vehicle (Mean ± SEM) | GFP+Bay (Mean ± SEM) | SP+Bay (Mean ± SEM) | P-Value^a^ | P-Value^b^ |
| --- | --- | --- | --- | --- | --- | --- |
| MUC5AC intensity in airways (arbitrary unit) | 25.485 ± 4.211 | 32.638 ± 4.725 | 30.637 ± 4.724 | 29.824 ± 4.724 | 0.009 | 0.802 |
| MUC5B intensity in airways (arbitrary unit) | 26.822 ± 2.082 | 25.459 ± 3.939 | 21.617 ± 2.677 | 23.035 ± 4.782 | 0.542 | 0.736 |
| *IL10* mRNA abundance (arbitrary unit) | 1.067 ± 0.182 | 0.879 ± 0.228 | 1.116 ± 0.295 | 1.145 ± 0.194 | 0.026 | 0.874 |
| IL10 protein concentration (pg/mL) in spent media | 20.432 ± 0.433 | 19.321 ± 0.394 | 19.460 ± 0.510 | 19.856 ± 0.503 | 0.011 | 0.398 |
| ^a^Paired T-test GPF-vehicle vs. SP-vehicle  ^b^Paired T-test GFP-Bay vs. SP-Bay |  |  |  |  |  |  |
